# Identification of a novel genetic locus underlying tremor and dystonia

**DOI:** 10.1186/s40246-017-0123-5

**Published:** 2017-11-06

**Authors:** Dorota Monies, Hussam Abou Al-Shaar, Ewa A. Goljan, Banan Al-Younes, Muna Monther Abdullah Al-Breacan, Maher Mohammed Al-Saif, Salma M. Wakil, Brian F. Meyer, Khalid S. A. Khabar, Saeed Bohlega

**Affiliations:** 1 Department of Genetics, King Faisal Specialist Hospital, and Research Centre, PO Box 3354, Riyadh, 11211 Saudi Arabia; 2 Saudi Human Genome Program, King Abdulaziz City for Science and Technology, Riyadh, Saudi Arabia; 3 Department of Neurosciences, King Faisal Specialist Hospital and Research Centre, PO Box 3354, Riyadh, 11211 Saudi Arabia; 4 Biomolecular Medicine, Research Centre, King Faisal Specialist Hospital and Research Centre, Riyadh, Saudi Arabia

**Keywords:** Dystonia, Tremor, Familial, Syndromic

## Abstract

**Background:**

Five affected individuals with syndromic tremulous dystonia, spasticity, and white matter disease from a consanguineous extended family covering a period of over 24 years are presented. A positional cloning approach utilizing genome-wide linkage, homozygozity mapping and whole exome sequencing was used for genetic characterization. The impact of a calmodulin-binding transcription activator 2, (*CAMTA2*) isoform 2, hypomorphic mutation on mRNA and protein abundance was studied using fluorescent reporter expression cassettes. Human brain sub-region cDNA libraries were used to study the expression pattern of *CAMTA2* transcript variants.

**Results:**

Linkage analysis and homozygozity mapping localized the disease allele to a 2.1 Mb interval on chromosome 17 with a LOD score of 4.58. Whole exome sequencing identified a G>A change in the transcript variant 2 5′UTR of *CAMTA2* that was only 6 bases upstream of the translation start site (c.-6G > A) (NM_001171166.1) and segregated with disease in an autosomal recessive manner. Transfection of wild type and mutant 5′UTR-linked fluorescent reporters showed no impact upon mRNA levels but a significant reduction in the protein fluorescent activity implying translation inhibition.

**Conclusions:**

Mutation of *CAMTA2* resulting in post-transcriptional inhibition of its own gene activity likely underlies a novel syndromic tremulous dystonia.

**Electronic supplementary material:**

The online version of this article (10.1186/s40246-017-0123-5) contains supplementary material, which is available to authorized users.

## Background

Currently, classification of dystonia relies upon phenomenology rather than etiology. A natural bifurcation is made based upon whether, with the exception of tremor, it is the sole motor feature (isolated dystonia) or is accompanied by other movement disorders including myoclonus (combined dystonia) [1]. Isolated dystonia may be subdivided into generalized or focal/segmental forms, with combined dystonia further categorized based upon the presence of myoclonus or Parkinsonism [2]. The advent of next generation sequencing has led to rapid expansion of phenotypic and genotypic subsets of syndromes primarily characterized as isolated or combined dystonias with more than 20 loci (DYT1-DYT27; some are redundant) identified to date [2, 3].

Familial-isolated dystonias are predominantly inherited as incompletely penetrant autosomal dominant traits most frequently related to the DYT1 or DYT6 loci with mutations in *TOR1A* [4, 5] or *THAP1* [6, 7], respectively. Additional isolated dystonia loci include DYT23 (*CIZ1*) [8], DYT24 (*ANO3*) [9], and DYT25 (*GNAL*) [10, 11], all of which have recently been extensively reviewed [2]. DYT4 (*TUBB4A*) may present as an autosomal dominant disorder in adolescence or early adulthood with prominent laryngeal dysphonia and craniocervical, segmental, or generalized dystonia [12, 13]. A rare recessively inherited generalized isolated dystonia results from mutation of *PRKRA* (DYT16) and is characterized by early onset and frequent association with dystonia-Parkinsonism [2, 14, 15]. Two other recessively inherited isolated dystonias have also been described arising from mutation of DYT2 (*HPCA*) [16] or DYT27 (*COL6A3*) [15]. Like these, other dystonia loci such as DYT23 (*CIZ1*) and DYT24 (*ANO3*) are yet to be independently confirmed. Dystonic movements are also associated with mutations of *ADCY5*, identified as the likely cause of a novel movement disorder, familial dyskinesia with facial myokimia (FDFM) [17, 18].

We previously reported a consanguineous extended family having a novel autosomal recessive syndromic tremulous dystonia with spasticity and white matter disease [19]. In this study, we further delineate and genetically characterize the syndrome, identifying *CAMTA2* as a novel candidate gene for a syndromic tremulous dystonia, and describe its clinical course and prognosis over a long follow-up period.

## Results

**Fig. 1:**
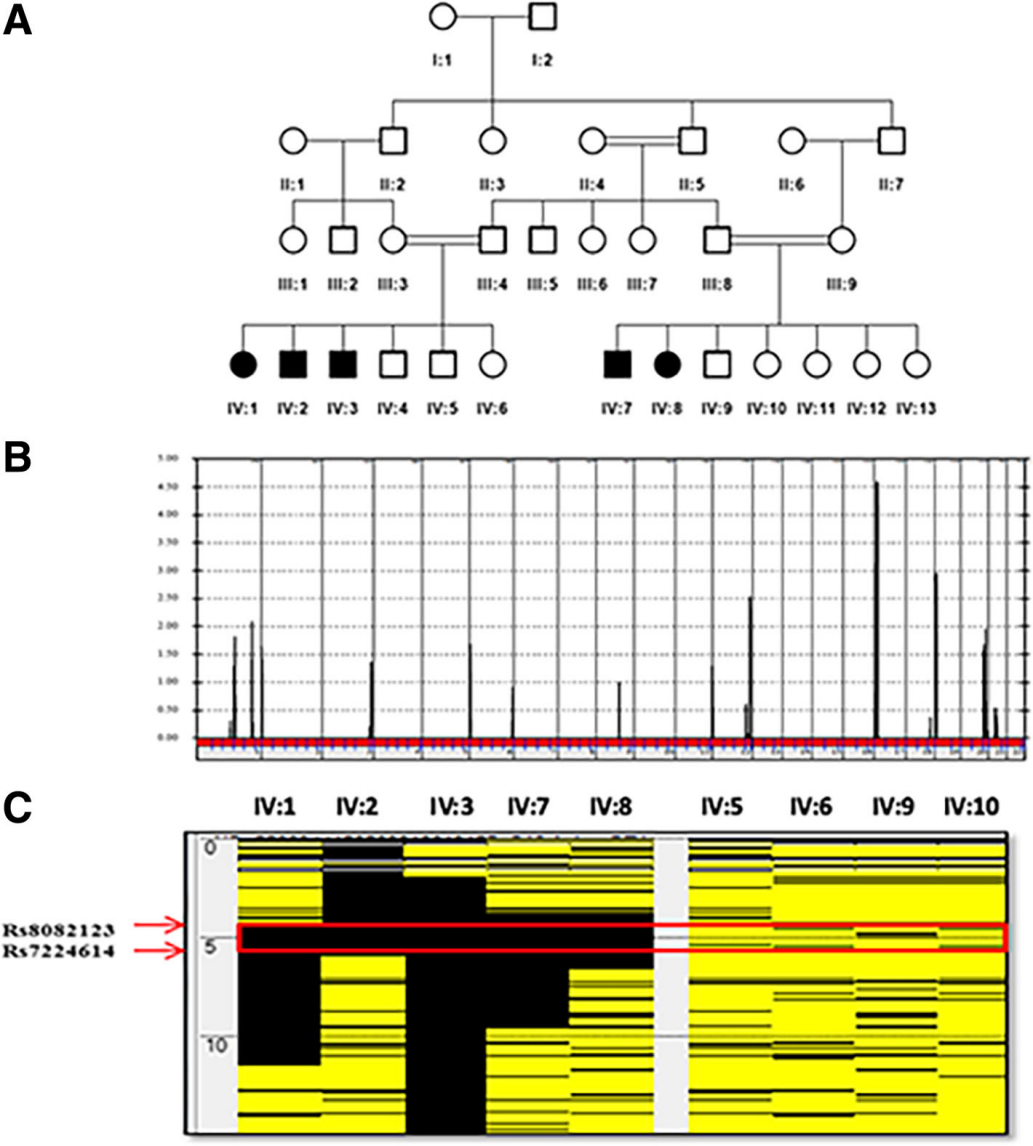
Identification of a dystonia-associated locus on chromosome 17. **a** Pedigree of an extended family with novel syndromic familial tremulous dystonia. **b** Genome-wide linkage analysis revealed a maximal peak with a LOD score of 4.58 on chromosome 17. **c** AutoSNPa output for chromosome 17 reveals an ROH (boxed in red) shared among affected members (IV:1, IV:2, IV:3, IV:7, and IV:8) and not present in unaffected individuals (IV:4, IV:5, IV:6, and IV:9)

The families originated from the Eastern part of the Arabian Peninsula. Five patients were studied (Fig 1a); their current ages ranging from 33 to 46 years. The detailed clinical description was previously reported [19]. In summary, the disease onset was at 7–8 years of age with tremulous movement in fingers and arms that progressed to affect the face, neck, and trunk. Speech became dysarthric and tremulous. The volitional movement was exacerbated by posture and action. Initially, gait was not impaired. Intelligence, personality, and memory were not affected apart from paranoia and depression in two individuals. Patients exhibited a coarse generalized tremor of 4–5 Hz, present at rest, waxing, and waning in amplitude with side-to-side (“no no” type tremor). In addition, there was variable focal or segmental dystonia with retrocollis shoulder elevation and hyperextension of the fingers. There was generalized hyperreflexia with extensor planter response noted around the age of 15 or 16 years. Sensory examination was intact and there was no truncal ataxia. Patients were followed for up to 24 years with no noticeable increase in their movement disorder. The patients were cognitively intact, they were able to finish college, hold independent jobs, get married, and have children. However, all of them exhibited variable degrees of spasticity affecting their legs more than arms (Additional file 1) and two of them required walking aids in their 30’s. Biochemical, organic acid, and lysosomal enzyme studies were normal. Visual and somatosensory evoke potentials showed evidence of prolonged central latencies. Accelerometric recording from the outstretched hand showed 4-Hz tremors. MRI of the brain showed similar abnormalities in all. There was mild symmetrical and confluent white-matter abnormalities sparing the U-fibers and involving the white matter of centrum semiovale, internal capsule, and ventral pons. These findings are not characteristic in any of the classic leukodystrophies. No change was observed in subsequent MRI scans with more than 20 years of follow-up. MR spectroscopy showed no lactate peak or other abnormalities. Patients failed to respond to therapeutic trials of ethanol or anticholinergics. However, some symptomatic benefits were noted with high-dose propranolol, gabapentin, and clonazepam.Additional file 1:Patient video. Patients exhibited a coarse generalized tremor of 4–5 Hz, present at rest, waxing and waning in amplitude with side-to-side (“no no” type tremor). In addition, there was variable focal or segmental dystonia with retrocollis shoulder elevation and hyperextension of the fingers. Patients exhibited generalized hyperreflexia with an extensor planter response being noted. (MOV 39038 kb).

Linkage analysis of the nuclear families with three and two affected individuals (Fig. 1a) resulted in independent LOD scores of 2.53 and 2.05, respectively, at rs11651665. Combined analysis (Fig. 1b) localized the disease to a region on chromosome 17 defined proximally by rs743646 and distally by rs34811366 (chr17:4,536,241-6,670,705) with a maximum LOD score of 4.58. Based upon runs of homozygosity shared by all the affected and absent in unaffected individuals (Fig. 1c; red rectangle), we refined the critical interval to a 2.1 Mb region (chr17:4,231,133-6,018,243) containing approximately 60 RefSeq genes (hg19).

**Fig. 2:**
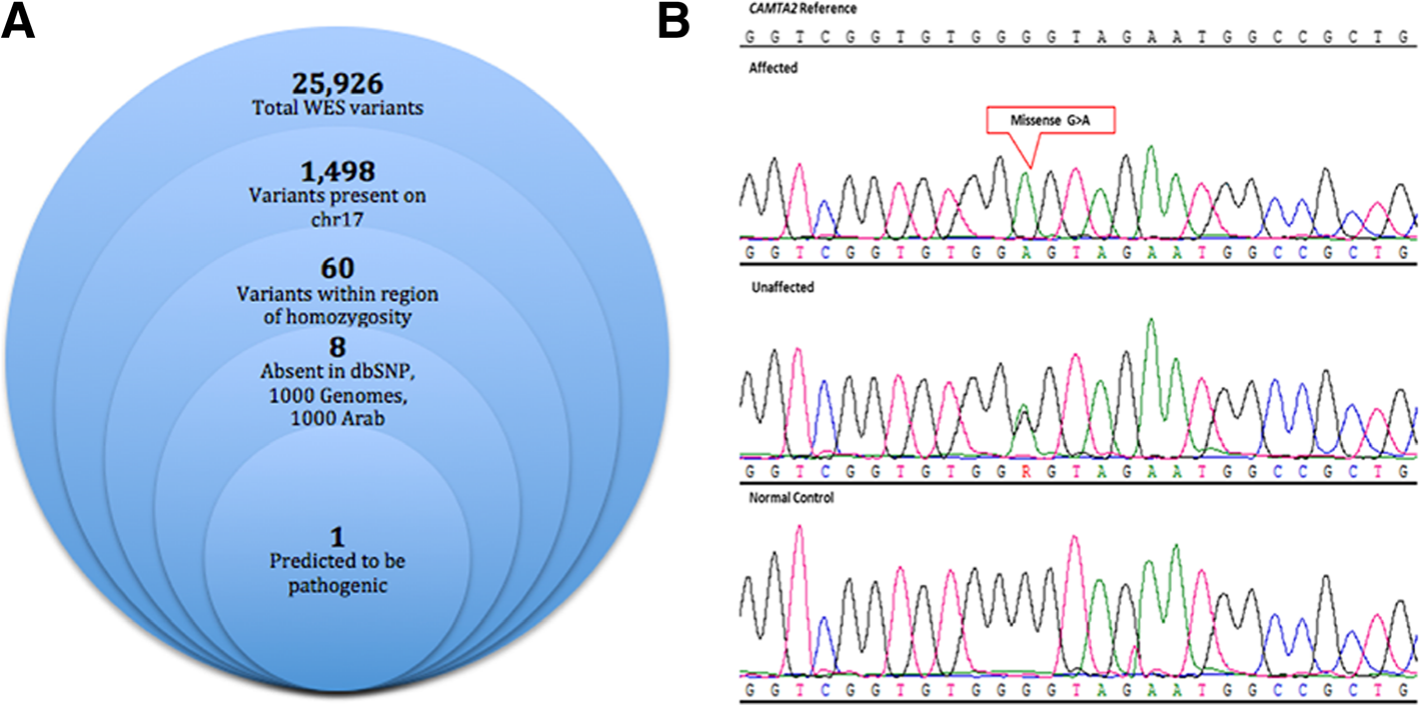
**a** Filtering strategy used for identification of a causative mutation in the syndromic familial tremulous dystonia patient. **b** DNA electrophoregram with the G>A change in 5′UTR (chr17:4890930) of the *CAMTA2* gene (reverse strand)

**Fig. 3:**
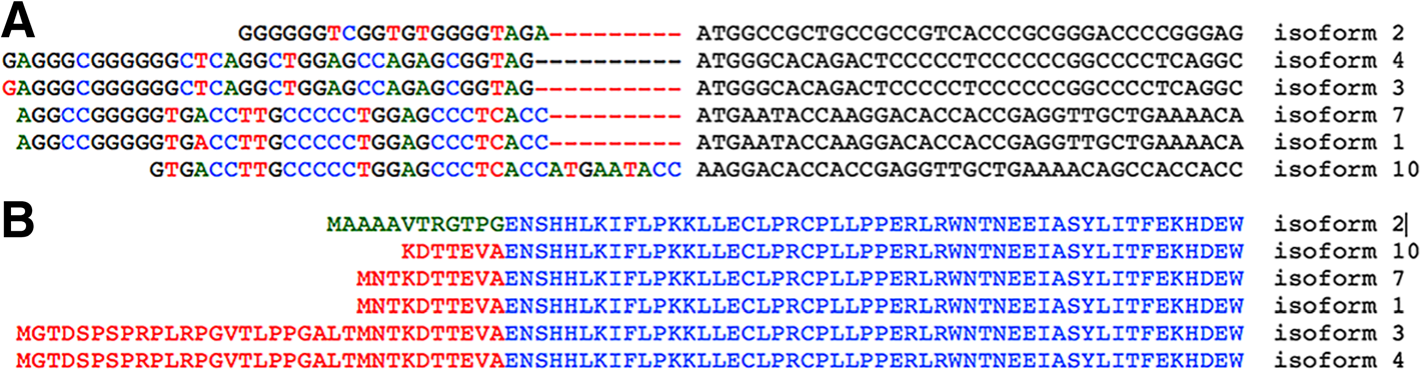
**a** Alignment of the nucleotide sequences of the six protein coding transcript variants of *CAMTA2*. **b** Amino acid alignment of the six isoforms of CAMTA2

Whole exome sequencing of individual IV:7 (Fig. 1a) identified 25,926 variants relative to the hg19 reference sequence, with 1498 variants on chr 17. These were further filtered to exclude all variants present outside our defined ROH (chr 17: 4,231,133-6,018,243) within which we identified 60 variants (Additional file 2). Within the critical interval (chr 17: 4,231,133-6,018,243), 99.42% of bases in the CDS and flanking regions of genes were covered at 30× or better with an average coverage of 425×. By excluding previously reported variants (present in dbSNP with incidence > 0.02; present in 1000 genomes with incidence > 0.02) and retaining those present at a frequency < 2% in more than 1000 Arab exomes held in an in-house database, the list was narrowed to 8. By only focusing on homozygous changes annotated as non-synonymous, splicing variants, frameshift insertions or deletions, and nonsense variants, we decreased the number of candidate variants to 1. It was a G>A change in the 5′UTR of transcript variant 2 of *CAMTA2* (c.-6G>A) (NM_001171166.1) (Fig. 2a). This homozygous variant was identified in 56 reads, was confirmed by Sanger sequencing (Fig. 2b), and segregated with disease in the extended family. *CAMTA2* has many transcript variants only six (1, 2, 3, 4, 7, and 10) of which are protein coding (https://www.proteinatlas.org/ENSG00000108509-CAMTA2/tissue). The aligned cDNA sequence of the translation start site and adjacent 5′UTR of these six variants show considerable variation (Fig. 3a). The resulting amino terminus amino acid sequences of these transcript variants are aligned and show that the first 12 amino acids of transcript variant 2 are not shared by the other protein coding transcript variants (Fig. 3b). Despite the absence of linkage or homozygozity mapping data to suggest involvement of any known dystonia loci, WES of all affected individuals, parents, and unaffected siblings from both nuclear families was undertaken. There were no variants in any of the known dystonia genes (*TOR1A*, *TUBB4A*, *THAP1*, *CIZ1*, *ANO3*, *GNAL*. *SCGE*, *GCH1*, *TH*, *TITF1*, *TAF1*, *PRKRA*, *ATP1A3*, *SLC63*, *ADCY5*) that survived filtration that removed alleles present in public databases at a frequency > 2%, in any affected individual.

**Fig. 4:**
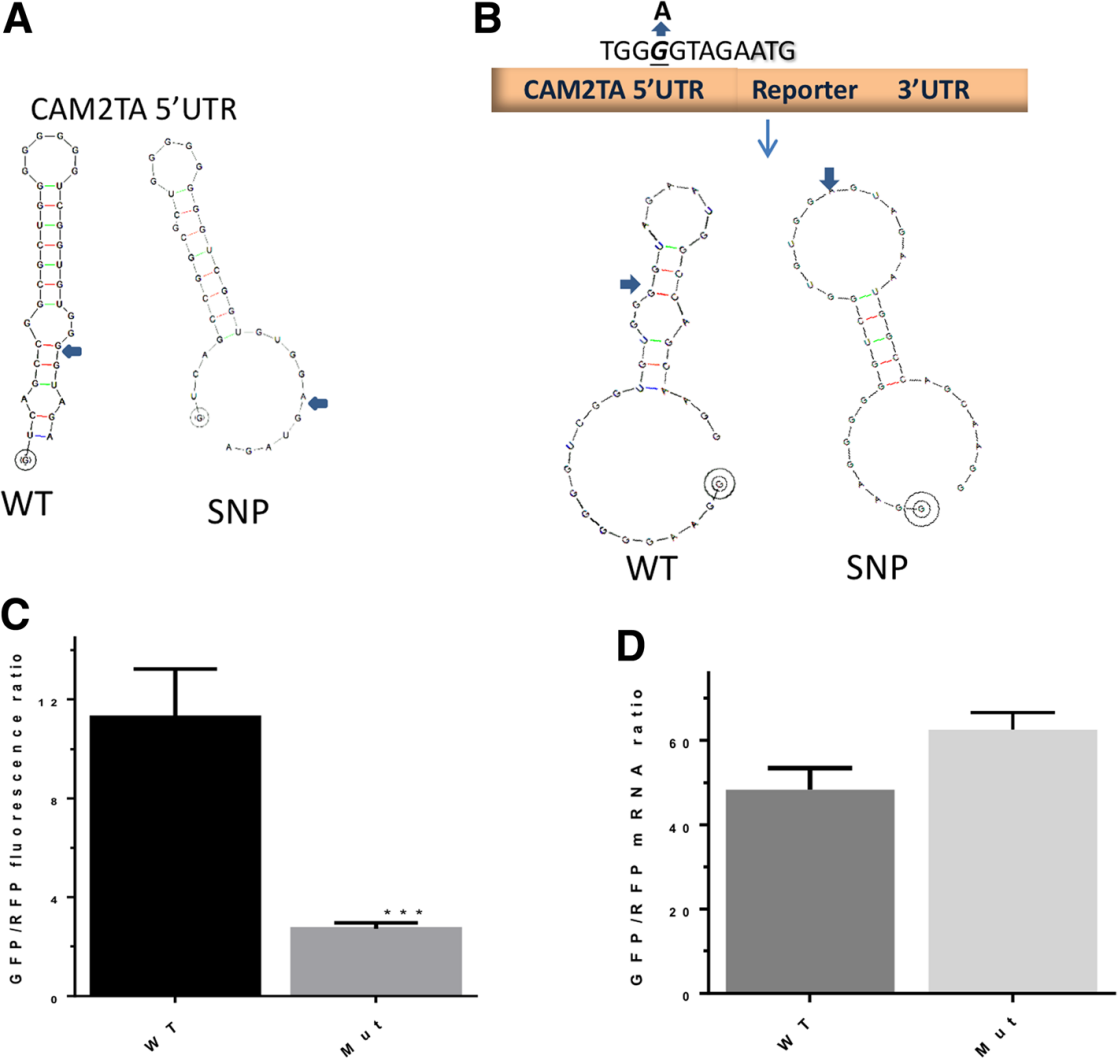
SNPs nucleotide sequence variation-mediated translational inhibition of CAMTA2. **a** Predicted secondary structures for wild type and mutant initiation region of CAMTA2. **b** Schematic diagram showing the wild type and mutant containing 5′UTR reporter constructs. **c** HEK293 cells (3 × 10^4^ cells/well) in 96-well plates were transfected with 75 ng of purified wild-type and mutant CAMTA2 5′UTR SGFP-expressing constructs along with control RFP expressing constructs (30 ng). After 20 h, fluorescence was quantified using automated image segmentation and quantification as described in the “Methods” section. The GFP/RFP expression ratio of WT and Mut of 5′UTR are shown as mean ± SD (*n* = 6). ***p < 0.0001 (Student’s *t* test). **d** Real-time qRT-PCR for reporter mRNA levels. HEK293 cells were transfected with wild type and mutant *CAMTA2* 5′UTR reporters for 24 h. Total RNA was extracted, and qRT-PCR was performed using specific primers for SGFP and RFP as described in the “Methods” section. Data are presented as the mean ± SD of two independent experiments. **p = 0.0014 (Student’s *t* test)

The wild-type *CAMTA2* 5′UTR 35 nt translation initiation region was subjected to analysis by a RNAdraw secondary structure program [20]. The structure obtained is a hairpin with three stems that is weakly stable (ΔG=11.1 kcal). The G>A variant 6 bases upstream of the *CAMTA2* transcript variant 2 initiation AUG changed this structure to a more relaxed form (ΔG=9.5 kcal) compared to the wild type. More importantly, the mutant variant secondary structure has only two stems and a wider loop as opposed to the three stem-tight loop of the wild type (Fig. 4a).

In order to evaluate the effects of the *CAMTA2* transcript variant 2 mutation on 5′UTR-linked activities such as transcription and translation, we constructed a fluorescence reporter cassette that contained a 25 nt initiation region upstream of SGFP (Fig. 4b). We subsequently transfected HEK293 cells with the wild type or mutant *CAMTA2* transcript variant 2 5′UTR initiation region containing expression cassettes and measured both the mRNA (qRT-PCR) and protein levels (fluorescence). We observed significant reduction in protein (fluorescence levels; (Fig. 4c)) but not mRNA levels of the mutant *CAMTA2* transcript variant 2 expression cassette relative to its wild-type counterpart (Fig. 4d).

**Fig. 5:**
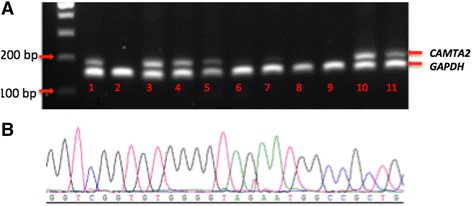
Expression of *CAMTA2* transcript variant 2 in sub-regions of human brain. **a** PCR amplification of *CAMTA2* transcript variant 2 and *GAPDH* (housekeeping gene) in (1) vermis cerebella, (2) amygdala, (3) cerebellum left, (4) cerebellum right, (5) cerebral cortex, (6) corpus callosum, (7) hippocampus, (8) occipital lobe, (9) frontal lobe, (10) olfactory lobe, and (11) cerebellar peduncles. **b**. Sanger sequencing electrophrogram showing cDNA sequence uniquely identifying *CAMTA2* transcript variant 2

PCR amplification of each protein coding isoform of *CAMTA2* indicated differential expression of isoform 2 which was only detected in the vermis cerebellum, left and right cerebellum, cerebral cortex, olfactory lobe, and cerebellar peduncles. Expression of this variant was not detected in the corpus callosum, hippocampus, occipital, or frontal lobes (Fig. 5a). GAPDH expression was used as an internal control for PCR amplification and specificity for each isoform confirmed by Sanger sequencing of amplicons as shown for transcript variant 2 (Fig. 5b). Other protein coding transcript variants of CAMTA2 (1, 3, 4, 7, and 10) were ubiquitously expressed in most sub-regions of the brain tested (Additional file 3).

## Discussion

We have described the clinical and genetic characteristics of a novel autosomal recessive familial tremulous dystonia syndrome, characterized by juvenile onset tremulous dystonia with spasticity and white matter disease. Phenotypically, the syndrome was heterogeneous within the families studied with all affected individuals displaying variable forms of both focal or segmental dystonia and coarse, asymmetrical, generalized tremor involving the head, neck and extremities [19]. Isolated dystonia is a classification reserved for exclusive occurrence of dystonia with the exception of tremor, whereas combined dystonia includes additional symptoms such as myoclonus or Parkinsonism by which it is further sub-classified [2].

Disease in the families we report is best characterized as a novel syndromic tremulous dystonia. The phenotype that combines dystonia, tremor, spasticity, and white matter disease clearly defines a novel clinical syndrome [19]. The novel nature of this syndrome is highlighted if not confirmed by identification of a previously undescribed single gene disorder segregating in an autosomal recessive manner within the families studied (LOD score 4.58; chr17:4,536,241-6,670,705). Whole exome and Sanger sequencing identified a homoallelic hypomorphic mutation (c.-6G>A) (NM_001171166.1) just upstream of the translation initiation site in the 5′UTR of *CAMTA2* transcript variant 2, with which disease segregated in the family studied. However, given the nature of WES where 100% coverage of the target region is almost never achieved and the coverage of only flanking regions (10–20 bp) of coding sequence in this study, and despite supporting functional evidence, we cannot exclude that a coding point mutation, CNV, deep intronic, or other mutation in the critical interval, in tight linkage disequilibrium with the reported variant, is the true causative mutation.

CAMTA proteins include multiple functional amino acid domains that are evolutionarily conserved including nuclear localization signals, nuclear export signals, a unique DNA binding domain CG-1, TIG-1 involved in protein dimerization, and ANK(ankyrin) repeats involved in protein-protein interactions [21–24]. CAMTA proteins also include a variable number of IQ motifs consisting of a repetitive sequence IQXXXRGXXX known to be associated with binding of Calmodulin (CaM) and CaM-like proteins. Transcription activation domains (TADS) have been reported in Arabadopsis AtCAMTA1, human HsCAMTA2, and functional support derived from reporter genes downstream of TAD’s in plant protoplasts or mammalian cell cultures [25]. Cardiac hypertrophy as a consequence transcriptional reprogramming of cardiac gene expression by CAMTA2 interaction with class II histone deacetylase has been reported [26]. In drosophila, CAMTA is highly expressed in retinal cells and has been shown to induce dFbx14 that deactivates the G-protein coupled receptor (GPCR) rhodopsin. This is essential to maintaining Ca^2+^ homeostasis of the photo-transduction pathway, a central mechanism of which is light induced Ca2+ influx and efflux regulated through CaM and dmCAMTA [27]. It is not surprising that *CAMTA2* is strongly expressed in both developing and adult murine retina; however, its function in this context remains unknown. Given the diverse expression and activity of GPCRs, it is reasonable to speculate that regulation of this class of molecule by CAMTA may modulate cellular growth and differentiation as evidenced in cardiac hypertrophy [26]. Both CAMTA1 and CAMTA2 are highly expressed in the heart and brain [27]. CAMTA2 has six protein coding isoforms (CAMTA2 ENSG00000108509) ranging from 178 to 1241 amino acids. The expression pattern and function of each isoform remains unknown.

In this study, we identified a G>A change in the 5′UTR of *CAMTA2* transcript variant 2. The position of the variant, near a splice site (+ 30) and adjacent to the translation initiation site (− 6) suggested that it might impact *CAMTA2* transcript variant 2 expression or translation. Our results clearly indicated that the *CAMTA2* mutant affected processes downstream of mRNA transcription, most likely translation as protein levels were dramatically reduced while there was no effect on mRNA levels (Fig. 4c–d). As protein expression was severely impacted without any alteration in the coding region (i.e, no amino acid change), the hypomorphic nature of the underlying mutation is clearly evident. It is known that secondary structures in the 5′UTR with stable high-energy forms (ΔG<-50 kcal/mol) inhibit translation if near the mRNA methyl G cap [28]. Hairpin structures of weaker energy coupled with positions far away from the 5’end cap such as in our case, in contrast, enhance translation [28]. The wild-type *CAMTA2* 5′UTR-predicted structure is near the initiation codon and of weakly stable structure, thus may facilitate translation machinery entry and therefore enhances translation. These activities are lost in the case of the mutant variant. In general, mutations near initiation regions can dramatically affect translation. Context sequence upstream of the initiation codon can modulate the scanning activity of the 40S ribosomal subunit affecting translation; mutations in this region, such as those in the Kozak consensus sequence that direct ribosomes to initiate protein synthesis, negatively affect translation initiation [29].

Further support for the pathogenicity or association of the *CAMTA2* variant with the phenotype described (for which we propose the name Bohlega Syndrome) in affected members of the family studied comes from the expression pattern of *CAMTA2* transcript variant 2 in human brain sub-regions where it is expressed in the vermis cerebellum, left and right cerebellum, cerebral cortex, olfactory lobe, and cerebellar peduncles (Fig. 5a). Expression of this isoform was not detected in the corpus callosum, hippocampus, and occipital or frontal lobes (Fig. 5a). Other CAMTA2 protein coding transcripts were ubiquitously expressed in all brain sub-regions tested. In mouse brain, CAMTA2 is expressed in Purkinje cells and granule cells of the dentate gyrus, pyramidal layer, and cerebellum (Additional file 4), but the distribution of each isoform is not known. Dystonia and Parkinsonism, including tremor, have largely been attributed to dysfunction of the basal ganglia. However, more recently, the role of Purkinje cells, the cerebellum, and their links to the basal ganglia in dystonia and Parkinson’s disease have been questioned [30–32]. Anatomical studies have identified connections from the basal ganglia and cerebellum via the thalamus to the cortex [33]. Of note, the ability of CAMTA2 knockout mice to mount a hypertrophic response to cardiac stress such as aortic banding is severely compromised [27]. There was no report of any tremor or dystonia in the CAMTA2 knockout mouse. In the affected individuals from our study, absence of any clinical cardiac symptoms extends the apparent discordance between the human and mouse phenotypes, albeit the mouse phenotype was induced by severe stress (aortic banding) [27]. However, the mouse ENCODE consortium mapped transcription factor binding and other regulatory domains across the mouse genome and compared them with the human genome. While they identified substantial conservation, they also identified a large degree of divergence between mouse and man in transcription and transcription regulation [34]. Therefore, differences in mouse and human phenotypes associated with genes such as *CAMTA2* involving transcription or transcription regulation should not be unexpected.

## Conclusions

Our study identifies a novel dystonia locus *CAMTA2* that opens a new line of investigation associated with the role of CAMTA2 in the growth, differentiation, survival, and function of cells and their cellular mechanisms within the brain as related to movement disorders. It also highlights the value of rare inherited disorders in providing insight into more common disease states such as dystonia and tremor.

## Methods

### Subjects and nucleic acid extraction

Five patients from a consanguineous extended family (two nuclear families) (Fig. 1a) were examined in the Department of Neurosciences, King Faisal Specialist Hospital and Research Center (KFSHRC). They were diagnosed with familial tremulous dystonia. All subjects were enrolled under a KFSHRC IRB-approved (RAC# 2090011) protocol with full-written informed consent.

### Genomic DNA extraction

Genomic DNA was extracted from peripheral blood samples of patients and family members using a Gentra Puregene Kit (QIAGEN GmbH, Hilden, Germany). Purity and quantity of DNA samples were assessed spectrophotometrically and stored at − 20 °C until required.

### Clinical data and imaging

Patients were seen and examined in the neurology clinic of King Faisal Specialist Hospital and Research Centre (KFSHRC) by certified neurologists (H.A. and S.B.). Briefly, the clinical data was obtained from the charts of patients at the time of the study and during the 20 years of follow-up. Imaging was performed by magnetic resonance imaging (MRI) 3 T using the following sequences: T1, T2, FLAIR, DWI, ADC, and MR spectroscopy. The other clinical, neurophysiological, and laboratory methodology may be reviewed from the previously published clinical paper [19].

### Linkage and Homozygosity mapping

All participating individuals (affected and unaffected) were genotyped using an Affymetrix Axiom Genome-Wide CEU array containing 587,353 markers (Affymetrix, Santa Clara, CA, USA) following the manufacturer’s protocol (http://www.affymetrix.com/support/technical/manuals.affx). Resulting genotypes were analyzed for shared runs of homozygosity (ROH) using autoSNPa (http://dna.leeds.ac.uk/autosnpa/). Linkage analysis was performed using the Allegro module of Easy Linkage assuming autosomal recessive inheritance and 100% penetrance (http://genetik.charite.de/hoffmann/easyLINKAGE/index.html). For linkage analysis, the extended family was broken down to reflect the two consanguineous nuclear families, with both independent LOD scores and a combined total LOD score being calculated, in a single multipoint analysis of both nuclear families.

### Whole exome sequencing and analysis

DNA was amplified to obtain a whole exome Ion Proton AmpliSeq library which was further used for emulsion PCR on an Ion One Touch System followed by an enrichment process using an Ion OneTouch ES, both procedures following the manufacturer’s instructions (Life Technologies, Carlsbad, CA, USA). The template-positive Ion PI Ion Sphere particles were processed for sequencing on the Ion Proton instrument (Life Technologies, Carlsbad, CA, USA). Approximately 15–17 Gb of sequence was generated per sequencing run. Reads were mapped to UCSC hg19 (http://genome.ucsc.edu/) and variants identified using the Ion Torrent pipeline (Life Technologies, Carlsbad, CA, USA). Based upon the consanguineous inbred pedigree and a clear autosomal recessive inheritance pattern in the family studied, the resultant variant caller file (vcf) was filtered to include only homozygous variants in the critical interval on chromosome identified by linkage and homozygosity mapping, within the pre-determined ROH shared by all affected individuals only. These were further filtered to only retain variants that were present in public databases including NHLBI Exome Sequencing Project, 1000 genomes project (phase 3), Exome Aggregation Consortium (ExAC) (version 0.3.1), dbSNP (build 141), and Kaviar (Known VARiants) (September 2015) databases at an incidence of < 2%. Included variants were further selected based upon pathogenicity predicted by Polyphen2 (http://genetics.bwh.harvard.edu/pph2/), SIFT (http://sift.jcvi.org/), and CADD (http://cadd.gs.washington.edu/info). Potential causative variant(s) were validated by Sanger sequencing and further vetted for familial segregation based upon autosomal recessive inheritance. For validation, an amplicon incorporating the variant of interest was amplified and sequenced using a BigDye Terminator kit (Applied Biosystems, Foster City, CA) and run on an ABI 3730xl automated sequencer (Applied Biosystems, Foster City, CA). SeqScape v.2.6 software (Applied Biosystems, Foster City, CA) was used to align sequence data and confirm variants. *CAMTA2* transcript variant 2 is a human isoform and aligns with the human reference sequence of this gene.

### Expression constructs

The *CAM2TA* 5′UTR reporter expression constructs were generated by using a cloning-free PCR method [35]. The *CAM2TA* 5′UTR wild type and mutant sequences (underlined) were incorporated in the forward primer: CCTATCAGTGATAGGGCGGCTGGG*TATATAA*TGGAAGGGGGGTCGGTGTGGG/AGTAGAATGGCCAGCAAGG. The sequence also contained a minimal promoter (TATA box in italics), 15 bases of complementary sequence to an expression vector containing strong modified EGFP, *UWr*-EGFP (SGFP), and a 3’UTR [36]. The reverse primer is complementary to a downstream region of the poly(A) site on the vector. Briefly, PCR was performed with the following conditions: 2.5 units of HotStart Taq(Qiagen) and 0.2 units of Pfx polymerase (Invitrogen, Carlsbad, CA) mix, 100 ng, template and cycle conditions: 95 °C for 12 min, 31 cycles of: 94 °C, 1 min., 55 °C, 1 min., 72 °C, 4 min., and final extension at 72 °C for 7 min. The PCR products were purified using Qiagen PCR purification columns and eluted in sterile water. The PCR products were run on a 1.2% agarose gel and visualized by ethidium bromide under UV light to verify size and quality. The resultant purified constructs contained a minimal promoter, *CAM2TA2* 5′UTR, SGFP reporter, and 3’UTR, and were used for transfection.

### Cells and transfection

HEK293 was obtained from the ATCC (Rockville, MD) and cultured in DMEM medium (Invitrogen, Carlsbad, CA) supplemented with 10% FBS and antibiotics at 37 °C in 5% CO_2_. Cells were seeded at 3 × 10^4^ cells per well in 96-well clear-bottom black plates (Matrix technologies, Hudson, NH) and incubated overnight. Cells were transfected with purified reporter constructs. Transfections with *CAM2TA2 5′UTR*-SGFP reporter along with a normalization control RFP expression plasmid driven by constitutive yet non-inducible RPS30 promoter [37] were performed in serum-free medium for 4 h, using Lipofectinamine 2000 (Invitrogen) according to the manufacturer’s instructions. Subsequently, the cell culture were replaced with completed DMEM medium and incubated further for 16 h. At these conditions, no effect on cell viability was observed. All transfections were performed in several replicates as indicated in the text. Fluorescence were acquired by the BD imaging pathway and quantified using the Proxcell imaging segmentation and quantification program [38]. The variance in GFP fluorescence among replicate microwells was < 10%. Data are presented as mean values ± standard deviation (SD) of total fluorescence intensity in each well, with at least six replicate readings.

### Real-time RT-PCR

Quantitative real-time (qRT-PCR) was performed for SGFP and RFP reporter mRNA using SybrGreen Real-Time PCR. Briefly, total RNAs were treated with DNase I, and cDNAs were used with Lightcycler Faststart DNA master SYBR Green I, (Roche, USA). The following primers were used: SGFP forward: GCCAGGTCTTTTCTGCAGTCACCG; reverse: GTAGTGCAGATGAACTTCAGG); human GAPDH forward: GGCAAATTCAACGGCACAGT; reverse: GATGGTGATGGGCTTCCC. In all cases, relative quantification of expression was determined using the standard curve method and the values obtained were within both detectable and linear range. Average concentrations were normalized to the endogenous GAPDH gene. The final results were converted to ratio ± SD of the specific mRNA levels to housekeeping mRNA levels. qRT-PCR was performed in multiplex using the Chroma 4DNA Engine thermocycler (Bio-Rad).

### Amplification of *CAMTA2* protein coding isoforms from human cDNA libraries

We used cDNA libraries prepared from total RNA isolated from normal human adult brain tissues: vermis cerebelli, amygdala, cerebellum left, cerebellum right, cerebral cortex, corpus callosum, hippocampus, occipital lobe, frontal lobe, olfactory, and cerebellar peduncles (BioChain Institute, Newark, CA) to determine the expression pattern of CAMTA2 isoforms in human brain sub-regions, skeletal muscle, and cardiac muscle. The cDNA libraries were used as template for PCR amplification of protein coding *CAMTA2* isoforms (isoform 1, isoform 2, isoform 3, isoform 4, isoform 7, and isoform 10). Briefly, PCR mixtures containing 2.5 ng cDNA, 1× PCR buffer, 2.5 mM each dNTP, 0.5 μM each primer, and 0.25 U HotStar Taq polymerase (Qiagen) were cycled 30 times at 95 °C for 15 min, 95 °C for 45 s, 64 °C for 45 s, and 72 °C for 60 s. *GAPDH* was amplified in a multiplex reaction as an internal PCR control. Primer information is available on request. The resulting amplicons were evaluated by electrophoresis on a 2% agarose gel. Representative bands were sequenced to confirm amplicon specificity.

## Additional files

Additional file 2: Variants in candidate interval. (PDF 217 kb)

Additional file 3: Expression of CAMTA2 isoforms, 1, 3, 4, 7 and 10 in sub-regions of the brain. (PDF 594 kb)

Additional file 4: CAMTA2 expression in brain. (PDF 1628 kb)
