# Supplementary material for: Identification of a novel genetic locus underlying tremor and dystonia
Source: Hum Genomics. 2017 Nov 6;11:25. doi: 10.1186/s40246-017-0123-5 (PMC5674688; doi:10.1186/s40246-017-0123-5)
Supplement: Supplementary file 2 — Variants in candidate interval. (PDF 217 kb) [file 40246_2017_123_MOESM2_ESM.pdf]

Identification of a novel genetic locus underlying tremor and dystonia.

Dorota M. Monies, PhD1,2, Hussam Abou Al-Shaar, MD3, Ewa A. Goljan, MSc1,2, Banan Al-Younes, MSc1,2, Muna Monther Abdullah Al-Breacan, BSc1, Maher Mohammed Al-Saif, BSc4, Salma M. Wakil, PhD1,2, Brian F. Meyer, PhD1,2, Khalid S. A. Khabar, PhD4 and Saeed Bohlega, MD2,3.

Movement Disorders

[1]Department of Genetics, Research Centre, King Faisal Specialist Hospital & Research Centre, Riyadh, Saudi Arabia

[2]Saudi Human Genome Program, King Abdulaziz City for Science and Technology, Riyadh, Saudi Arabia

[3]Department of Neurosciences, King Faisal Specialist Hospital & Research Centre, Riyadh, Saudi Arabia.

[4]Biomolecular Medicine, Research Centre, King Faisal Specialist Hospital & Research Centre, Riyadh, Saudi Arabia

Correspondence:
Dr. Saeed Bohlega, MD, FRCPC
Department of Neurosciences
King Faisal Specialist Hospital & Research Centre
PO Box 3354, Riyadh 11211
Kingdom of Saudi Arabia
Tel: +966-11-4424760
Fax: +966-11-4424763
boholega@kfshrc.edu.sa

VARIANTS IN CRITICAL INTERVAL

chromosome	start pos	refGene gene	refGene function	knownGene AA change
chr17	4350182	SPNS3	splicing	
chr17	4352636	SPNS3	exonic	SPNS3:uc002fxu.3:exon6:c.496G>A:p.G166R
chr17	4455500	MYBBP1A	exonic	MYBBP1A:uc002fxz.4:exon7:c.825A>G:p.A275A
chr17	4457116	MYBBP1A;MYBBP1A	exonic;splicing	MYBBP1A:uc002fxz.4:exon5:c.550A>G:p.I184V
chr17	4461748	GGT6;GGT6	exonic	GGT6:uc002fyc.4:exon3:c.948C>T:p.P316P
chr17	4497160	SMTNL2	exonic	SMTNL2:uc002fye.2:exon4:c.342T>C:p.Y114Y
chr17	4536241	ALOX15	exonic	ALOX15:uc002fyh.3:exon11:c.1455A>G:p.T485T
chr17	4542794	ALOX15	exonic;splicing	ALOX15:uc002fyh.3:exon2:c.268G>C:p.D90H
chr17	4574751	PELP1	exonic	PELP1:uc002fyi.4:exon17:c.3376A>T:p.T1126S
chr17	4575283	PELP1	exonic	PELP1:uc002fyi.4:exon16:c.3003T>C:p.P1001P
chr17	4575923	PELP1	exonic	PELP1:uc002fyi.4:exon16:c.2363T>G:p.V788G
chr17	4575936	PELP1	exonic	PELP1:uc002fyi.4:exon16:c.2350T>C:p.S784P
chr17	4576347	PELP1	exonic	PELP1:uc002fyi.4:exon16:c.1939A>C:p.T647P
chr17	4576620	PELP1	exonic	PELP1:uc002fyi.4:exon15:c.1770G>A:p.P590P
chr17	4620497	ARRB2	splicing	
chr17	4641755	CXCL16	exonic	CXCL16:uc002fyr.4:exon3:c.306G>A:p.G102G
chr17	4645788	ZMYND15	splicing	
chr17	4684095	TM4SF5;TM4SF5	exonic;splicing	TM4SF5:uc002fyw.1:exon2:c.189G>A:p.P63P
chr17	4693054	GLTPD2;GLTPD2	exonic;splicing	BC150535:uc002fyz.2:exon1:c.337C>T:p.P113S
chr17	4699552	PSMB6	exonic	PSMB6:uc002fzb.4:exon1:c.63G>A:p.A21A
chr17	4714199	PLD2	exonic	PLD2:uc002fzc.3:exon10:c.963C>T:p.H321H
chr17	4718776	PLD2;PLD2	exonic;splicing	PLD2:uc002fzc.3:exon13:c.1179G>A:p.E393E
chr17	4720469	PLD2	exonic	PLD2:uc002fzc.3:exon17:c.1730C>T:p.T577I

chr17	4722876	PLD2;PLD2	exonic;splicing	PLD2:uc002fzc.3:exon23:c.2461G>A:p.G821S
chr17	4796274	MINK1;MINK1	exonic;splicing	MINK1:uc010vsp.2:exon14:c.671T>C:p.V224A
chr17	4796286	MINK1;MINK1	exonic;splicing	MINK1:uc010vsp.2:exon14:c.683C>T:p.P228L
chr17	4796561	MINK1	splicing	
chr17	4797305	MINK1	exonic	MINK1:uc010vsp.2:exon17:c.970G>A:p.V324I
chr17	4837300	GP1BA	exonic	GP1BA:uc021tob.1:exon1:c.1401T>C:p.S467S
chr17	4849030	PFN1	UTR3	
chr17	4849193	PFN1	UTR3	
chr17	4890930	CAMTA2	UTR5	
chr17	4904564	KIF1C	exonic	KIF1C:uc002gan.2:exon5:c.231T>C:p.I77I
chr17	4910308	KIF1C	exonic	KIF1C:uc002gan.2:exon14:c.1264_1265AG
chr17	4923876	KIF1C	exonic	KIF1C:uc002gan.2:exon20:c.1840C>A:p.P614T
chr17	4926882	KIF1C	exonic	KIF1C:uc002gan.2:exon23:c.2748A>G:p.P916P
chr17	4936898	SLC52A1	exonic	SLC52A1:uc010ckw.3:exon2:c.520G>A:p.V174M
chr17	4937575	SLC52A1	exonic	SLC52A1:uc002gao.4:exon3:c.209A>G:p.Q70R
chr17	5036272	USP6;USP6	exonic;splicing	USP6:uc002gav.1:exon5:c.263A>C:p.K88T
chr17	5042894	USP6	exonic	USP6:uc002gav.1:exon14:c.1423T>C:p.W475R
chr17	5085857	ZNF594	exonic	ZNF594:uc021tol.1:exon1:c.1695G>A:p.A565A
chr17	5086163	ZNF594	exonic	ZNF594:uc021tol.1:exon1:c.1388_1389insA:p.S463fs
chr17	5280440	RABEP1	exonic	RABEP1:uc010cld.1:exon13:c.1926T>C:p.R642R
chr17	5284698	RABEP1;RABEP1	exonic;splicing	RABEP1:uc002gbl.4:exon16:c.2286A>G:p.Q762Q
chr17	5284719	RABEP1	exonic	RABEP1:uc002gbl.4:exon16:c.2307T>C:p.N769N
chr17	5284770	RABEP1	exonic	RABEP1:uc002gbl.4:exon16:c.2358G>A:p.R786R
chr17	5289580	NUP88;NUP88	exonic;splicing	NUP88:uc002gbo.2:exon17:c.2172T>C:p.H724H
chr17	5290033	NUP88;NUP88	exonic;splicing	NUP88:uc002gbo.2:exon16:c.2157A>G:p.K719K
chr17	5290703	NUP88	splicing	
chr17	5294976	NUP88;NUP88	exonic;splicing	NUP88:uc002gbo.2:exon10:c.1389A>T:p.P463P

chr17	5308377	NUP88;NUP88	exonic;splicing	NUP88:uc002gbo.2:exon6:c.1044G>A:p.T348T
chr17	5326089	RPAIN;RPAIN	exonic;splicing	RPAIN:uc002gbp.1:exon3:c.413C>T:p.A138V
chr17	5326145	RPAIN;RPAIN	exonic;splicing	RPAIN:uc002gbp.1:exon3:c.469C>G:p.P157A
chr17	5326162	RPAIN	splicing	
chr17	5347576	DHX33	exonic	DHX33:uc010clf.3:exon8:c.1578C>T:p.Y526Y
chr17	5392820	MIS12	UTR3	
chr17	5461878	NLRP1	exonic	NLRP1:uc002gcg.1:exon4:c.2138C>A:p.P713Q
chr17	5487164	NLRP1	exonic	NLRP1:uc002gcg.1:exon1:c.114G>C:p.S38S
chr17	5991344	WSCD1	exonic	WSCD1:uc031qyk.1:exon2:c.114C>T:p.H38H
chr17	6014176	WSCD1	exonic	WSCD1:uc010clj.3:exon5:c.168A>G:p.T56T
