# Supplementary material for: Identification of a novel genetic locus underlying tremor and dystonia
Source: Hum Genomics. 2017 Nov 6;11:25. doi: 10.1186/s40246-017-0123-5 (PMC5674688; doi:10.1186/s40246-017-0123-5)

Identification of a novel genetic locus underlying tremor and dystonia

#Dorota Monies, PhD[1,2], Hussam Abou Al-Shaar, MD[3], Ewa A. Goljan, MSc[1,2], Banan Al-Younes, MSc[1,2], Muna Monther Abdullah Al-Breacan, BSc[1], Maher Mohammed Al-Saif, BSc[4], Salma M. Wakil, PhD[1,2], Brian F. Meyer, PhD[1,2], Khalid S. A. Khabar, PhD[4] and #Saeed Bohlega, MD[2,3].

[1]Department of Genetics, Research Centre, King Faisal Specialist Hospital & Research Centre, Riyadh, Saudi Arabia

[2]Saudi Human Genome Program, King Abdulaziz City for Science and Technology, Riyadh, Saudi Arabia

[3]Department of Neurosciences, King Faisal Specialist Hospital & Research Centre, Riyadh, Saudi Arabia.

[4]Biomolecular Medicine, Research Centre, King Faisal Specialist Hospital & Research Centre, Riyadh, Saudi Arabia

#These authors contributed equally.

Dorota Monies: moniesdm@gmail.com
Hussam Abou Al-Shaar: aboualshaar.hussam@gmail.com
Ewa A. Goljan: enaim@kfshrc.edu.sa
Banan Al-Younes: Bal-yousef@kfshrc.edu.sa
Muna Monther Abdullah Al-Breacan: Malbreacan@kfshrc.edu.sa
Maher Mohammed Al-Saif: mahersaif@kfshrc.edu.sa
Salma M. Wakil: smajid@kfshrc.edu.sa
Brian F. Meyer: meyerb@kfshrc.edu.sa
Khalid S. A. Khabar: khabar@kfshrc.edu.sa
Saeed Bohlega: boholega@kfshrc.edu.sa

Correspondence:

Dr. Saeed Bohlega, MD, FRCPC
Department of Neurosciences
King Faisal Specialist Hospital
& Research Centre
PO Box 3354, Riyadh 11211
Kingdom of Saudi Arabia
Tel: +966-11-4424760
Fax: +966-11-4424763
boholega@kfshrc.edu.sa

Dr. Dorota Monies, PhD
Department of Genetics
King Faisal Specialist Hospital
& Research Centre
PO Box 3354, Riyadh 11211
Kingdom of Saudi Arabia
Tel: +966-11-4424263
Fax: +966-11-4424763
moniesdm@gmail.com

Expression of CAMTA2 isoforms, 1, 3, 4, 7 and 10 in sub-regions of the brain.

CAMTA2 protein isoforms (1, 3, 4, 7 and 10) were ubiquitously expressed in most sub-regions of the brain tested.

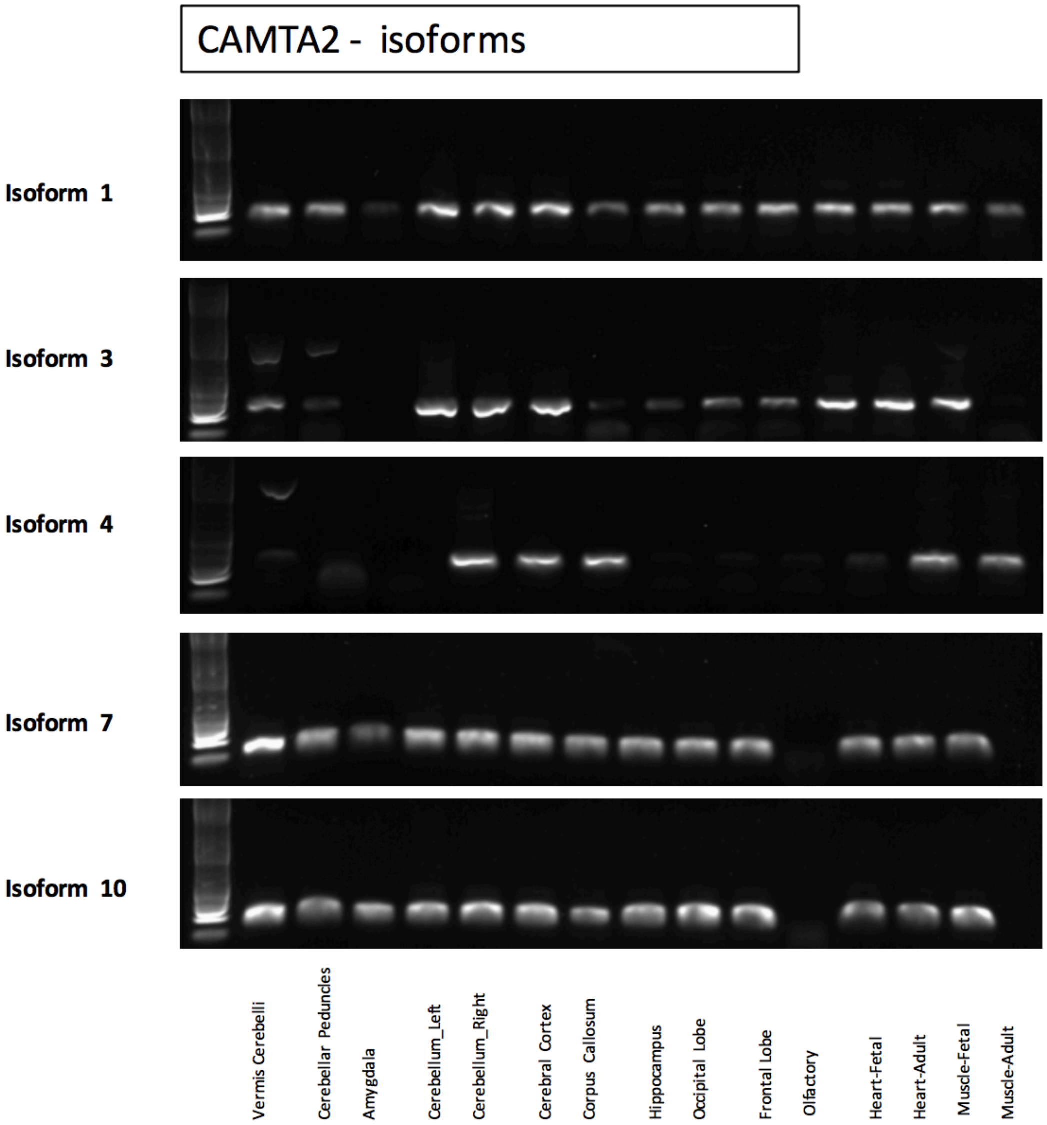

Supplement: Supplementary file 3 — Expression of CAMTA2 isoforms, 1, 3, 4, 7 and 10 in sub-regions of the brain. (PDF 594 kb) [file 40246_2017_123_MOESM3_ESM.pdf]
