# Supplementary material for: Identification of a novel genetic locus underlying tremor and dystonia
Source: Hum Genomics. 2017 Nov 6;11:25. doi: 10.1186/s40246-017-0123-5 (PMC5674688; doi:10.1186/s40246-017-0123-5)
Supplement: Supplementary file 4 — CAMTA2 expression in brain. (PDF 1628 kb) [file 40246_2017_123_MOESM4_ESM.pdf]

Identification of a novel genetic locus underlying tremor and dystonia.

Dorota M. Monies, PhD1,2, Hussam Abou Al-Shaar, MD3, Ewa A. Goljan, MSc1,2, Banan Al-Younes, MSc1,2, Muna Monther Abdullah Al-Breacan, BSc1, Maher Mohammed Al-Saif, BSc4, Salma M. Wakil, PhD1,2, Brian F. Meyer, PhD1,2, Khalid S. A. Khabar, PhD4 and Saeed Bohlega, MD2,3.

Movement Disorders

[1]Department of Genetics, Research Centre, King Faisal Specialist Hospital & Research Centre, Riyadh, Saudi Arabia [2]Saudi Human Genome Program, King Abdulaziz City for Science and Technology, Riyadh, Saudi Arabia
[3]Department of Neurosciences, King Faisal Specialist Hospital & Research Centre, Riyadh, Saudi Arabia.
[4]Biomolecular Medicine, Research Centre, King Faisal Specialist Hospital & Research Centre, Riyadh, Saudi Arabia

Correspondence:
Dr. Saeed Bohlega, MD, FRCPC
Department of Neurosciences
King Faisal Specialist Hospital & Research Centre
PO Box 3354, Riyadh 11211
Kingdom of Saudi Arabia
Tel: +966-11-4424760
Fax: +966-11-4424763
boholega@kfshrc.edu.sa

In situ Hybridization

In situ hybridization images were downloaded from the Allen Institute for Brain Science at www.alleninstitute.org (Lein et al. 2007). Detailed methods used for preparation of slides, probe design, hybridization, staining, analysis and annotation are fully documented at http://help.brain-map.org/download/attachments/2818169/ABADataProductionProcesses.pdf?version=1&modificationDate=1319477 154403

Expression of CAMTA2 in human brain

Immunohistochemical (IHC) staining of P56 mouse brain sagittal sections revealed expression of CAMTA2 in the granule cell layers of the dentate gyrus and the pyramidal layer, and less strongly in the piriform area pyramidal layer (Figure Panel A). CAMTA2 was also expressed in the cerebral cortex, specifically the granular layer of lobules IV-V, the simple lobule, crus 1, crus 2, paramedian lobule and copula pyramidus. Significant expression was also noted in the dorsal cochlear nucleus and principal sensory nucleus of the trigerminal nerve. Relative expression of CAMTA2 is indicated by the heatmap (Figure Panel B). Intense staining of Purkinje cells in the cerebellum was also evident

(Figure Panel C). Expression patterns in mouse brain for specific isoforms of CAMTA2 were not available.

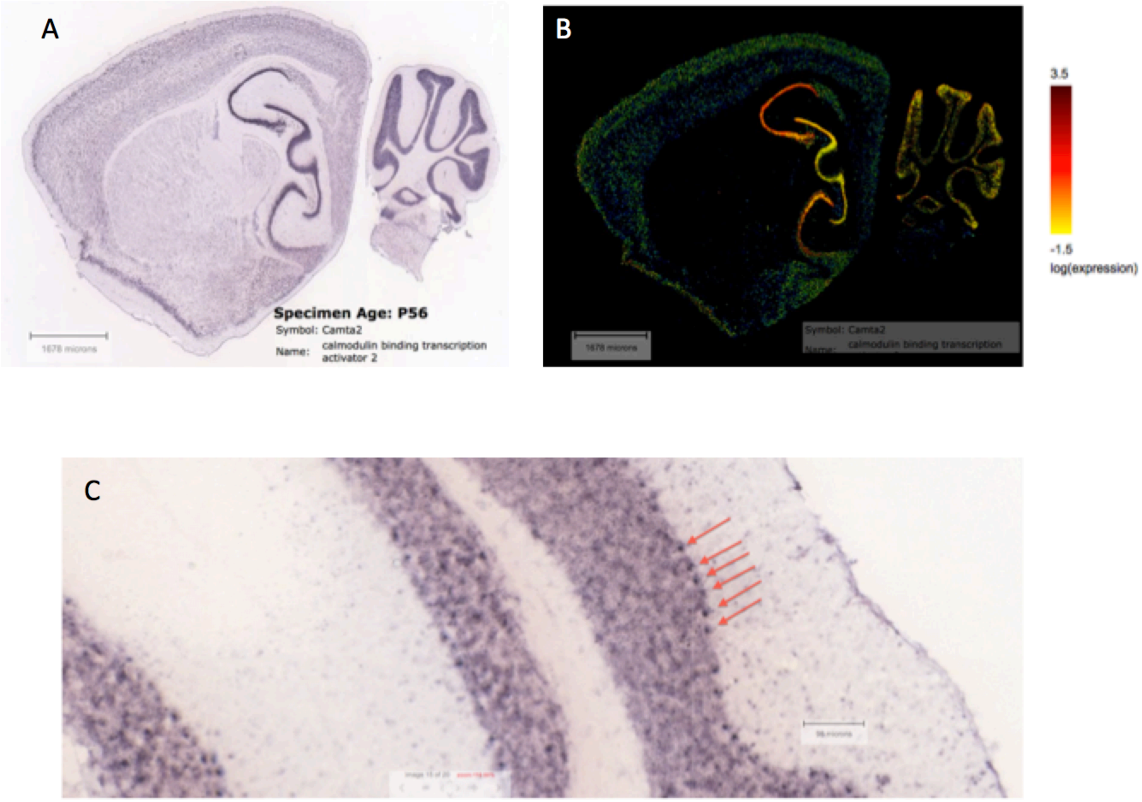
A
Specimen Age: P56
Symbol: Camta2
Name: calmodulin binding transcription activator 2
1678 microns
B
Symbol: Camta2
Name: calmodulin binding transcription
1678 microns
3.5
-1.5
log(expression)
C
